# Color Stability of Rhodium-Coated Archwire after Immersion in Mouthwashes

**DOI:** 10.1155/2023/7259012

**Published:** 2023-03-02

**Authors:** Sami Kadhum Al-Joubori, Sara M. Al-Mashhadany, Alaa Faleh Albo Hassan

**Affiliations:** Department of Orthodontics, College of Dentistry, University of Baghdad, Baghdad 00964, Iraq

## Abstract

**Background:**

Is to evaluate the color stability of rhodium-coated aesthetic archwires after immersion in two types of mouthwashes (fluoridated and nonfluoridated), study the effect of immersion time, and to compare the effect of these solutions on color stability of the aesthetic archwires.

**Materials and Methods:**

35 rhodium-coated aesthetic archwires were prepared and divided two halves, arranged in seven strips (each strip contains 10 wires) and immersed in Deionized water, Sidrazac and Biofresh solution. The measurements of the color have been carried out through a computed spectrophotometer based on Commission Internationale de I'Eclairage *L*^*∗*^*a*^*∗*^*b*^*∗*^system, and color variations (Δ*E*^*∗*^), color measurements were repeated 7 and 21 days after immersion in the solution. Statistical analyses include mean, standard deviation, minimum, maximum, and inferential statistics which include ANOVA and Tukey (HSD) for testing for any statistically significant differences in light reflection of the groups; *t*-test was used to test for differences in immersion time intervals. The significance level has been set at *P* ≤ 0.05.

**Results:**

Both types of mouthwashes resulted in color changes in different degrees and a higher color instability amount has been noted with Sidrazac-fluoridated mouthwashes, The color change amount has been increased with the time being statistically higher in 3 weeks of immersion, while there is nonsignificant color change after immersion in the Biofresh mouthwash.

**Conclusion:**

Rhodium-coated archwire shows high color changes in Sidrazac-fluoridated mouthwashes and nonsignificant color change after immersion in the Biofresh mouthwash.

## 1. Introduction

The need for aesthetic orthodontic equipment keeps growing, thus it is necessary to find materials that offer patients acceptable aesthetics and clinicians' suitable clinical performance [1]. Metallic archwires that have been coated with tooth-colored resin materials now represent the available solution to the aesthetic problems; ideally, the aesthetic archwire color must be matching the color of the natural teeth and aesthetic brackets. This is a recent development in orthodontic wires [2].

Clinically significant is color stability of aesthetic archwires that are used in orthodontic treatments; any discoloration, staining, or changes in the patient's appearance will affect willingness to cooperate and accept their treatment; color instability regarding such wires and the exposure of underlying metal are frequently reported as well. According to research, 25% of the coating is lost intraorally within a period of 33 days, which causes the wire to lose its aesthetic appeal [2].

The aesthetic archwires' discoloration may result from both external as well as internal factors, food dyes and colored mouthwashes could discolor the outside of objects, and coating material type and the roughness of its surface have a significant impact on how much discoloration is induced by certain substances [3]. Water absorption and oral hygiene are two examples of variables that may have an impact on the degree of color change [4].

The objective of the present study is to evaluate the color stability of rhodium-coated aesthetic archwires after immersion into two mouthwash types (fluoridated and non-fluoridated) and the effect of immersion time and to compare the effect of these solutions on color stability of the aesthetic archwire; the null hypothesis is that the mouthwash had no effect on the color stability of rhodium-coated aesthetic archwires.

## 2. Materials and Methods

The process and the protocol of this work have been approved by the Scientific Committee of College of Dentistry, University of Baghdad, in accordance with the Helsinki Declaration for the human search study (No. 672422).

### 2.1. The Samples

In this study, the sample size measured by post hoc power analysis using *G*^*∗*^Power (version 3.1.9.4, Win) for one-way ANOVA tests assuming *α* = 0.05 and a power of 0.80. Based on this assumption, a sensitivity analysis was carried out based on the anticipated sample size (*N* = 70, control = 10, *N*1 = *N*2 = *N*3 = 20), resulting in a minimum detectable effect size of Cohen's *d* = 0.379. This effect size was nearly similar to the previous study [3].

**Figure 1:**
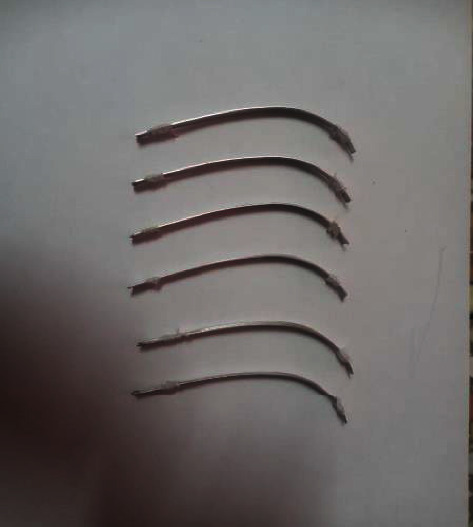
The sample strips.

Thirty-fiverhodium-coated aesthetic archwires (0.019^*∗*^0.025 NiTi, Fantasia wires) have been prepared, every one of the samples has been made through the cutting of preformed archwires to 2 halves so the sample became 70 wire, followed by placing each 10 halves of coated archwire segments together and uniting their free ends first by light cured composite resin due to the fact that it has a quick set, so that the sample arranged into seven strip (each strip contain ten) as shown in Figure 1, the first strip used for the baseline color measurement and each two strips (20 half wire) immersed in the following solution for one-week and three-week color measurement:

1. Deionized water
2. Biofresh (nonfluoridated mouth wash): contain 0.12% chlorhexidine digluconate, sodium saccharine, cremophor, purified water, flavor, and glycerin (Scitra Co, Sharjah, U.A.E)
3. Sidrazac (fluoridated mouthwash): contain 0.12% chlorhexidine digluconate, deionized water, sodium fluoride, menthol, and aroma (Alpha Pharma, Adana, Turkey)

### 2.2. Baseline Color Measurements

**Figure 2:**
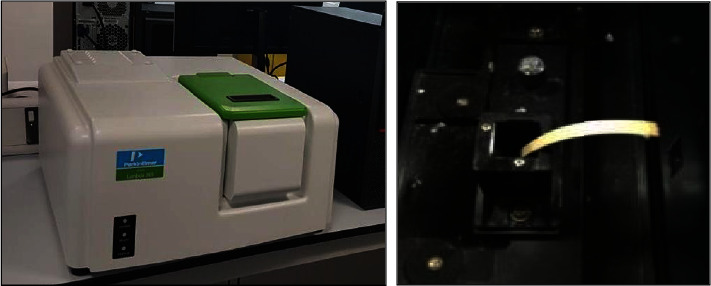
The specimen was centered in front of the xenon lamp in the center of a spectrophotometer's tube (Perkin Elmer, USA).

The top surfaces of the specimens always were centered in front of the xenon lamp (light source of spectrophotometer) in the center of a spectrophotometer's tube, so repetitive measurements for each specimen could be taken from the same specimen's region (Figure 2). With the use of a spectrophotometer (Perkin Elmer, Waltham, Massachusetts, United States), the color was evaluated based on the Commission Internationale de l'Eclairage's 1976 *L*^*∗*^*a*^*∗*^*b*′^*∗*^ color space system at the Ministry of Science and Technology in Baghdad. They were incubated in distilled water in glass containers at a temperature of 37°C for 24 hrs with the use of an incubator after numbering the specimens of each subgroup from 1 to 10 for Deionized, Biofresh, and Sidrazac solution with a marker pencil that could not be removed by solutions. Baseline measurements have been then done for measuring light reflection regarding every one of the specimens through the spectrophotometer at visible wave lengths starting from 400 nm–700 nm at intervals of 10 nm, which is whyfor every one of the specimens, we make thirty-one. The values for *X*, *Y*, and *Z* were collected, and the system was transformed to the CIE color space using the MATLAB 8 (version 8, R2012b, 2012/USA). 3D colorimetric measurements are used by the CIE system: the brightness of a color is represented by *L*^*∗*^ values, red-green content by *a*^*∗*^ values, and yellow-blue content by *b*^*∗*^ values, using the following equations [5]:

(1) $$\begin{array}{c} L*=116{\left(\frac{Y}{Y0}\right)}^{\left(1/3\right)}-16\text{,}\\ a*=500\left[{\left(\frac{X}{X0}\right)}^{\left(1/3\right)}-{\left(\frac{Y}{Y0}\right)}^{\left(1/3\right)}\right]\text{,}\\ b*=200\left[{\left(\frac{Y}{Y0}\right)}^{\left(1/3\right)}-{\left(\frac{Z}{Z0}\right)}^{\left(1/3\right)}\right]\text{.} \end{array}$$

where X, Y, Z tristimulus values previously measured, *X*0, *Y*0, *Z*0X, Y, Z values of a perfect white sample(standard), L*∗* CIE Lab *L* value (lightness in Lab color space), a*∗* CIE Lab *a* value (red-green value), and b*∗* CIE Lab *b* value (yellow-blue value).

### 2.3. Measurement of Color Changes

 Following immersion in solutions, samples have been put in a glass container along with prepared solutions (Deionized water, Biofresh, and Sidrazac), and the container was after that incubated in the incubator at a temperature of 37°C. The solution was changed twice daily at intervals of 12 hrs, and the color measurements have been repeated 7 (T1) and 21 days (T2) after the immersion. The samples have been removed out of the solution then rinsed by the distilled water for a duration of five mins before each measurement. The samples were given time to dry after excess water on surfaces has been removed away using a tissue paper. *L*^*∗*^, *a*^*∗*^, and *b*^*∗*^ values of every one of the specimens were afterwards determined following immersion in treatment solutions. The following color variation was observed between baseline measurements and those which made the following solution immersion:

(2) $$\begin{array}{c} ∆L*=L2\_L1\text{,}\\ ∆a*=a2–a1\text{,}\\ ∆b*=b2–b1\text{.} \end{array}$$

Then, total color differences Δ*E*^*∗*^ for every one of the specimens (i.e., the distance between 2 point in the color space) have been estimated based on the following equation:

(3) $$\begin{array}{c} ∆E*=\left(∆L*2+∆a*2+∆b*2\right)\frac{1}{2}. \end{array}$$

The Δ*E*^*∗*^ of each group was compared with others, to distinguish which solution leads to more color changes in the aesthetic archwires [6].

### 2.4. Pilot Study

Many trails had been done on the spectrophotometer to check that the readings are correct and the machine is working properly, and to ensure that the samples' width is enough to be scanned by the spectrophotometer and to ensure that the immersion time is good enough to make a color change in the aesthetic archwires in a way that it could be detected by the spectrophotometer.

### 2.5. Statistical Analyses

Statistical Package for the Social Sciences (SPSS) (version 23, SPSS Inc., Chicago, USA) is used for data analyses; the data are normally distributed according to Shapiro–Wilk's test, including the following:

1. Descriptive statistics: mean, standard deviation, minimum, and maximum
2. Inferential statistics: one-way ANOVA was used for testing for any statistically significant differences in light reflection of the groups; Tukey (HSD) was used to test for differences in two subgroups when ANOVA revealed a difference, and *t*-test was used to test for differences in immersion time intervals. The significance level for each statistical test has been set at *P* ≤ 0.05

## 3. Results

Tables 1 and 2 show the descriptive statistics for color change of aesthetic archwire and its difference among the three mouthwashes at each time interval and there is a statistically significant difference in the total color difference of rhodium-coated aesthetic archwires between Sidrazac and both Biofresh and distilled water after one week and three weeks of immersion using Tukey's (HSD) test.

**Figure 3:**
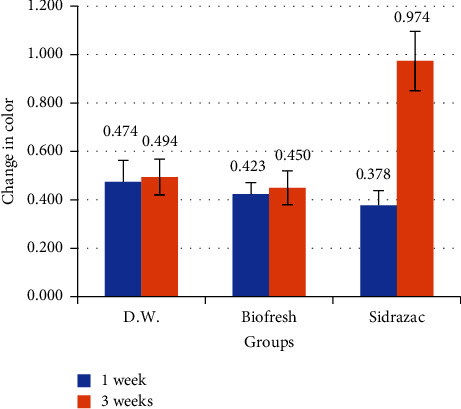
Color changes of rhodium-coated archwire after immersion in mouthwashes.

In comparison to Biofresh mouthwash, Sidrazac generated a much larger amount of color change over the course of three weeks (Table 3). The quantity of color change grew over time, yet the greatest change amount had occurred in the third week (Figure 3).

According to the findings of this research, the null hypothesis was rejected and the alternative one approved that the mouthwash had an effect on the color stability of rhodium-coated aesthetic archwires, and there is a statistically significant difference in the color difference of rhodium-coated aesthetic archwires between Sidrazac and both Biofresh and distilled water after one week and three weeks of immersion, in vitro.

## 4. Discussion

Throughout orthodontic treatment, coated aesthetic archwires' color stability is crucial. The color stability regarding aesthetic orthodontic appliances, like archwires, brackets, and ligatures, has been a subject of numerous color measurements' research works. The color regarding coated aesthetic archwires ought to be competitive with the aesthetic brackets, the color of the teeth, and the other aesthetic orthodontic components. On the other hand, the color of a person's natural teeth might vary depending on their gender, race, age, and visual perception. Instrumental measurements, such as spectrophotometers, are employed to gauge color stability of coated aesthetic archwires in order to get around issues with visual color comparison. CIE *L*^*∗*^*a*^*∗*^*b*^*∗*^ color space is the most extensively utilized and accepted method for measuring color because it is suitable for determining slight color changes [7].

Spectrophotometer has been used in many previous studies to measure color change by comparing the values of *L*^*∗*^*a*^*∗*^*b*^*∗*^ before and after treatment according to the CIE Lab system, but the value of Δ*E*^*∗*^ represents relative color changes that an observer might report for the materials after immersion. Thus, Δ*E*^*∗*^ is more meaningful than the individual *L*^*∗*^, *a*^*∗*^, *b*^*∗*^ values [8]. Values of Δ*E*^*∗*^ < *l* were regarded as not appreciable (perceptible) by the human eye. If 3.3 > Δ*E*^*∗*^ > *l*, this color difference is appreciable by a skillful operator but considered clinically acceptable. Whilst values of Δ*E*^*∗*^ > 3.3 are appreciable by nonskilled persons and considered clinically unacceptable [9].

Archwire with a rectangular cross section (0.019^*∗*^0.025 inch) was chosen because it is easier to make a strip, and the total width of each sample had to be at least 5 mm to provide a large surface area for proper color measurement [7].

Mouthwash is a good way to get rid of plaque and gingivitis, and people also use it for social and aesthetic reasons [10]. So, this study used two commercially available mouthwashes: Sidrazac with fluoride and Biofresh without fluoride. Both of these mouthwashes have moderate effects on plaque and some anti-inflammatory effects that help get rid of gingival inflammation [11].

In the current study, rhodium aesthetic archwires showed perceptible color change after 3 weeks of exposure to varying degrees of Sidrazac mouthwash. Contrary to the findings of earlier research by Mujawar et al. [12] and da Silva et al. [7], which found that aesthetic archwires displayed discernible color change following 21 days of the immersion in the staining solution, the quantity of color change increased with time [7, 12, 13] and is consistent with earlier research [3, 14, 16]. However, while dealing with the Biofresh mouthwash, Inami et al. [17] investigated color stability of aesthetic archwires and reported a minor change in the color that has been consistent with results of the current investigation.

According to a previous study on the color stability of various aesthetic archwires [18], there are variations in the rate of color change of various aesthetic archwires from various manufacturers under the same condition. In addition, a color change occurs in the same archwire when immersed in various mouthwashes [3]. These results are in line with those of Al-Attar [19], who discovered that mouthwash molecules diffuse and adsorb to the surface of ceramic and sapphire brackets, causing bracket discoloration when they are submerged in the mouthwash. This might be related to the physical and chemical constituents of the aesthetic archwires, the cause of which needs to be determined by further research.

The causes of aesthetic archwires' color differences is due to water absorption, the adsorption or absorption of colorants from mouthwashes. In addition, it was discovered that mouthwashes with high alcohol concentrations and low pH levels could damage the surface integrity regarding the coating material, leading to discoloration, and that color variations of aesthetic archwires could be associated with these factors [18, 20–22]. Color variations in the mouth may be caused by a variety of confounding factors, such as food variation, dental cleanliness, chewing frequency, and salivary composition. The aesthetic orthodontic 'archwires' variations in thickness and surface properties may also have an impact on the color measurements [23].

The readings of color change values (Δ*E*^*∗*^) increased gradually as the time of immersion increased, which is in agreement with Al-Attar [19] and Albo Hassan and Ghaib [22], who found that the amount of discoloration of aesthetic brackets was reported to increase as the time of immersion in mouthwashes and staining solutions increased. This is likely due to absorption or adsorption of colorant molecules of the mouthwashes to the coating material, which causes this material to deteriorate with time, superficial penetration of the mouthwashes, and chemical degradation of the material surface [22]. In addition, further studies are needed to test other important variables in the same conditions, such as nickel release [24] and cytotoxicity [25] of the rhodium-coated archwires.

## 5. Conclusions

Following a three-week immersion in Sidrazac-fluoridated mouthwashes, rhodium-coated archwire exhibits significant color changes, while there is a negligible color change following immersion in the Biofresh mouthwash, the quantity of color change increased over time.

## Figures and Tables

**Table 1:** Descriptive statistics for color change of aesthetic archwire and its difference among the three mouthwashes at each time interval.

Immersion time	Group	Descriptive statistics					Group differences	
		*N*	Mean	S.D	Min	Max	F-test	*P* value
1 week	D.W	13	0.474	0.089	0.32	0.58	6.499	0.004
	Biofresh	13	0.423	0.048	0.31	0.53		
	Sidrazac	13	0.378	0.060	0.31	0.51		

3 weeks	D.W	13	0.494	0.074	0.41	0.64	130.269	0.001
	Biofresh	13	0.450	0.070	0.33	0.61		
	Sidrazac	13	0.974	0.123	0.83	1.22		

**Table 2:** Tukey HSD for each two groups of mouthwashes.

	Groups		Mean difference	*P* value
1 week	D.W	Biofresh	0.051	0.153
		Sidrazac	0.096	0.003
	Biofresh	Sidrazac	0.045	0.219

3 weeks	D.W	Biofresh	0.044	0.451
		Sidrazac	−0.480	0.001
	Biofresh	Sidrazac	−0.524	0.001

**Table 3:** Color difference between time intervals of immersion in the three mouthwashes.

Groups	Descriptive statistics				Time difference	
	1 week		3 weeks			
	Mean	S.D	Mean	S.D	*t*-test	*P* value
D.W	0.474	0.089	0.494	0.074	−0.624	0.538
Biofresh	0.423	0.048	0.450	0.070	−1.146	0.263
Sidrazac	0.378	0.060	0.974	0.123	−15.742	0.001

## Data Availability

The raw data collected by the researcher from which the statistical analysis obtained was saved completely and can be forward any time under your request.

## Additional Points

The current study was conducted in an in vitro setting. An attempt has been made to simulate the oral environment, but it is always complex and cannot be accurately replicated in vitro.
